# Magnetic resonance imaging under isoflurane anesthesia alters cortical cyclooxygenase‐2 expression and glial cell morphology during sepsis‐associated neurological dysfunction in rats

**DOI:** 10.1002/ame2.12167

**Published:** 2021-05-03

**Authors:** Ibtihel Dhaya, Marion Griton, Jan Pieter Konsman

**Affiliations:** ^1^ INCIA Institut de Neurosciences Cognitives et Intégratives d'Aquitaine CNRS UMR 5287 Bordeaux France; ^2^ Univ. Bordeaux INCIA UMR 5287 Bordeaux France; ^3^ Laboratoire de Neurophysiologie Fonctionnelle et Pathologies UR/11ES09 Faculté des Sciences Mathématiques Physiques et Naturelles Université de Tunis El Manar Tunis Tunisie; ^4^ Service de Réanimation Anesthésie Neurochirurgicale Centre Hospitalier Universitaire (CHU) de Bordeaux Bordeaux France

**Keywords:** anesthesia, astrocyte, blood‐brain barrier, magnetic resonance imaging, microglia, sepsis

## Abstract

**Background:**

Magnetic resonance imaging (MRI) of rodents combined with histology allows to determine what mechanisms underlie functional and structural brain changes during sepsis‐associated encephalopathy. However, the effects of MRI performed in isoflurane‐anesthetized rodents on modifications of the blood‐brain barrier and the production of vasoactive prostaglandins and glia cells, which have been proposed to mediate sepsis‐associated brain dysfunction, are unknown.

**Methods:**

This study addressed the effect of MRI under isoflurane anesthesia on blood‐brain barrier integrity, cyclooxygenase‐2 expression, and glial cell activation during cecal ligature and puncture‐induced sepsis‐associated brain dysfunction in rats.

**Results:**

Cecal ligature and puncture reduced food intake and the righting reflex. MRI under isoflurane anesthesia reduced blood‐brain barrier breakdown, decreased circularity of white matter astrocytes, and increased neuronal cyclooxygenase‐2 immunoreactivity in the cortex 24 hours after laparotomy. In addition, it annihilated cecal ligature and puncture‐induced increased circularity of white matter microglia. MRI under isoflurane anesthesia, however, did not alter sepsis‐associated perivascular cyclooxygenase‐2 induction.

**Conclusion:**

These findings indicate that MRI under isoflurane anesthesia of rodents can modify neurovascular and glial responses and should, therefore, be interpreted with caution.

## 1 INTRODUTION

The past decades have seen the application of new experimental imaging approaches to study brain chemistry and structure in vivo. While some of the recent in vivo imaging techniques are still invasive and require a “cranial window” resulting in morphological activation of glial cells,^1^, ^2^ others like magnetic resonance imaging (MRI) are noninvasive. However, relatively few attempts have been made to compare the findings obtained with brain imaging to histological methods or to evaluate the effects of these new approaches on brain tissue with histological approaches. This is important, not because histology would be an artifact‐free gold standard, but simply because a large part of our knowledge of brain chemistry and structure has been obtained with histological approaches.

Although anesthesia is widely used in animal brain imaging to avoid stress and movement artifacts, both clinical and experimental evidence has accumulated indicating that anesthesia has effects on the central nervous system (CNS) beyond that of simply reducing neuronal activity (see for review Ref. [3). Indeed, given that MRI is noninvasive, several groups have been able to evaluate the influence of anesthesia by including a group of restrained awake animals. Thus, it has been shown that anesthesia affects cerebral hemodynamics and autoregulation.^4^, ^5^, ^6^, ^7^ But more recent work indicates that repeated restraint, necessary to habituate rats for awake functional brain scanning, can result in long‐lasting changes in stress responses.^8^ It is therefore likely that the use of anesthetics will continue to be part of the majority of in vivo MRI protocols.

For repeated or longitudinal MRI experiments, isoflurane anesthesia is recommended because it provides easy control, good recovery, and robust stimulus‐induced functional brain vascular responses.^9^ However, when it comes to cerebrovascular changes assessed with MRI in the absence of stimulation, isoflurane anesthesia results in patterns that are very different from that found in awake subjects.^10^, ^11^ In terms of tissue integrity, it has been shown that 3 hours of anesthesia, isoflurane results in less dysfunction of the blood‐brain barrier (BBB) in the cortex than sevoflurane.^12^ Thus the choice of the anesthetic agent should take into account the read‐outs of interest for a study involving brain MRI.

Based on its overall well‐known profile of effects compared to other anesthetics, our previous work employed isoflurane anesthesia to assess with MRI cerebral blood flow and perfusion as well as water diffusion in experimental models of bacterial sepsis‐associated neurological dysfunction.^13^, ^14^, ^15^ For the sepsis model consisting of the peripheral administration of bacterial lipopolysaccharide (LPS), it has been shown, among different anesthetics tested, that the early LPS‐induced changes in hemodynamics under isoflurane anesthesia were closest to those observed in awake rats.^16^ But isoflurane anesthesia does reduce LPS‐induced systemic as well as brain cell production of the pro‐inflammatory cytokine interleukin‐1β^17^, ^18^, ^19^, ^20^ and increases survival.^21^ Regarding the more pathophysiologically relevant cecal ligature and puncture (CLP) sepsis model, isoflurane, like sevoflurane, improves survival, and mitigates lung inflammation in rats.^22^ However, in mice, isoflurane has been shown to not affect, reduce or increase mortality.^23^, ^24^, ^25^, ^26^

Since we have recently shown that changes in perfusion and structural brain MRI after CLP in rats were associated with reduced neuronal expression of the prostaglandin‐synthesizing enzyme cyclooxygenase‐2 (COX‐2) expression and changes in glial cell morphology,^15^ we wondered what effect MRI under isoflurane anesthesia could have on histological measures of experimental sepsis‐associated brain dysfunction. In the present work, we therefore studied the effect of 2.5 hours of MRI under isoflurane anesthesia and CLP on immunohistochemical detection of BBB leakage, COX‐2 expression, and glial cell morphology.

## 2 MATERIALS AND METHODS

### 2.1 Animals

Experiments were conducted according to European recommendations on animal research (European Parliament and Council Directive of 22 September 2010 (2010/63/UE)) and reviewed by the local committee for animal experimentation (ASF‐SNC‐SEP DIR 36). Forty‐two male Wistar rats (Charles Rivers, L’Arbresle, France), weighing a mean 343.36 ± 2.35 g and 3.5 months old were housed two per cage in a temperature‐controlled room (22 ± 1.0°C) in a 12 hours dark‐light cycle with free access on water and food during one week before starting experiments. During this acclimation period, animals were left undisturbed except for daily handling starting three days before surgery.

### 2.2 Surgery, behavioral evaluation and magnetic resonance imaging

Twenty‐four rats were subjected under isoflurane anesthesia to polymicrobial intra‐abdominal infection induced by CLP as previously described.^15^, ^27^ In 18 sham‐operated rats, the same manipulations were performed except CLP and saline injection. At the end of the operation, animals received 5 mL of saline and analgesia (butrophanol, Torbugesic^®^; 2 mg/kg subcutaneously) and were placed in a clean individual cage. Thirty minutes after the end of surgery animals were awake and moving around. To study nonspecific sickness responses during sepsis, daily food and water intake, and body weight were measured at several time points prior and posterior to surgery. As described previously,^15^, ^28^ sham‐ and CLP‐operated rats were tested at different time points before and after surgery for two simple non‐postural (pinna and corneal reflexes) and one complex postural somatomotor reflex (righting reflex) to evaluate neurological dysfunction. Twenty‐four hours after sham‐ or CLP surgery, half of each rats group (12 CLP and 9 Sham) were anesthetized with isoflurane (induction 3%‐5%, maintenance 1.5%) for MRI. The other rats were left undisturbed, except for handling, weighing and reflex assessment, and did neither undergo anesthesia nor imaging the day after surgery. MRI experiments were performed on a 7 T horizontal‐bore scanner (Advance III console, Bruker, Ettlingen, Germany) and took, including preparation, less than three hours. Intracolonic temperature was constantly monitored and the animal was heated when colonic temperature dropped below 35.5°C by warm water circulating in the bed used to position the rat inside MRI scanner. Respiration was assessed with a ventral pressure sensor and heart rate recorded using an MRI compatible electrode.^15^ Isoflurane concentration was adapted to maintain respiratory rate around 50 per minute (±10). Cardiac frequency mean was about 410 beats per minute at onset and increased during anesthesia to reach a mean of 490.

### 2.3 Immunohistochemistry, microscopy, and image analysis

At the end of MRI or 27 hours after abdominal surgery, rats were deeply anaesthetized by intraperitoneal injection of 60 mg/kg of sodium pentobarbital. Brain tissue preparation, immunohistochemistry, and image acquisition and analysis were performed as previously described.^13^, ^14^, ^15^ Commercially available antisera raised against COX‐2 (goat anti‐COX‐2; M‐19 sc‐1747, lot# F2512, Santa Cruz Biotechnology, Heidelberg, Germany) diluted 1:750, the microglia‐macrophage‐specific ionized calcium‐binding adaptor molecule (Iba‐1; rabbit anti‐Iba‐1; 019‐19741, Wako Chemicals GmbH, Neuss, Germany) diluted 1:1000 and rat immunoglobulin G (IgG; biotinylated goat anti IgG, BA‐9401, Vector Laboratories, Burlingame, CA, USA) diluted 1:2000 and the intermediate filament glial fibrillary acidic protein GFAP (mouse anti‐GFAP mAb, clone GA5, MAB360, Merck Millipore, Fontenay sous Bois, France) diluted 1:500 were used.

In every brain section between bregma +4.85 and −2.85 mm, the occurrence and extent (scored as restricted [1], intermediate [2], or extensive [3]) of perivascular IgG diffusion in a given structure was noted. The cumulative score of the extent of IgG diffusion for a given brain region or structure was also divided by the number of sections that contained the region or structure in question. Quantification of COX‐2 labeling in the cortex was performed by applying a particle size criterion on 8‐bit images obtained after the application of a fixed threshold using Image J (http://imagej.nih.gov/ij/). The cortex was chosen for COX‐2 given that COX‐2‐mediated prostaglandin production is important in the regulation of cortical blood flow^29^, ^30^ and our previous finding that CLP decreased perfusion towards the cerebral cortex.^15^ For Iba‐1 and GFAP immunostaining, images of the center and lateral corpus callosum were analyzed as our previous findings showed increased axial water diffusion in the corpus callosum of the corpus callosum.^15^ Iba‐1‐immunopositive glia cells were characterized both in term of density and morphology using the Image J plugin Fraclac (http://rsb.info.nih.gov/ij/plugins/fraclac/fraclac.html) as previously described.^15^ Iba‐1 cell size and activation were also evaluated as described by Hovens and colleagues.^31^ Image editing software (Adobe Photoshop, Adobe Systems, San Jose, CA, USA) was used only to adjust contrast and brightness for photomicrographs composing illustrating figures.

### 2.4 Data representation and analysis

Data were expressed as means ± standard error of the mean (SEM). Food intake and water intake were expressed relative to 100 g of body weight and 100 g of food intake, respectively, and were analyzed with a one‐way ANOVA (CLP surgery as a between factor) except for neurological scoring where Mann‐Whitney *U* test was used. Image analysis data of immunohistochemically stained brain sections were analyzed by a two‐way ANOVA (CLP surgery and MRI under isoflurane anesthesia as between factors). When data were not normally distributed, a log10 or square root transformation was performed, and if still not normally distributed then the nonparametric Scheirer‐Ray‐Hare extension of the Kruskall‐Wallis test was considered. The Benjamini‐Hochberg procedure for multiple comparisons was used for the dozen brain regions in which perivascular IgG diffusion was assessed. Significant ANOVA effects were further analyzed by the Newman‐Keuls post hoc test. In all cases, a level of *P* < .05 was considered as statistically significant.

## 3 RESULTS

### 3.1 CLP induced sepsis‐ associated brain dysfunction

Within the 23 hours following the end of abdominal surgery, no mortality was observed in the sham‐operated group (one animal did die, however, at the induction of anesthesia for imaging) while 3 out of 24 CLP animals died (12.5% mortality). Mann‐Whitney *U* tests on food consumption relative to body weight during the 24 hours after the start of surgery showed a significant lower food intake in animals that underwent CLP as compared to sham‐operated rats (*U*: 67.0, *P* < .05). Mann‐Whitney tests on reflexes showed that CLP significantly reduced the righting reflex 4 hours (*U*: 102, *P* < .05), 8 hours (*U*: 24.5, *P* < .001) and 24 hours (*U*: 19.5, *P* < .001) with a trend to decrease the pinna reflex 4 hours later (*U*: 137.0, *P* = .054) as compared to sham surgery without affecting the corneal reflex (see Figure 2, ^15^).

### 3.2 MRI under anesthesia attenuates BBB breakdown following abdominal surgery

**FIGURE 1:**
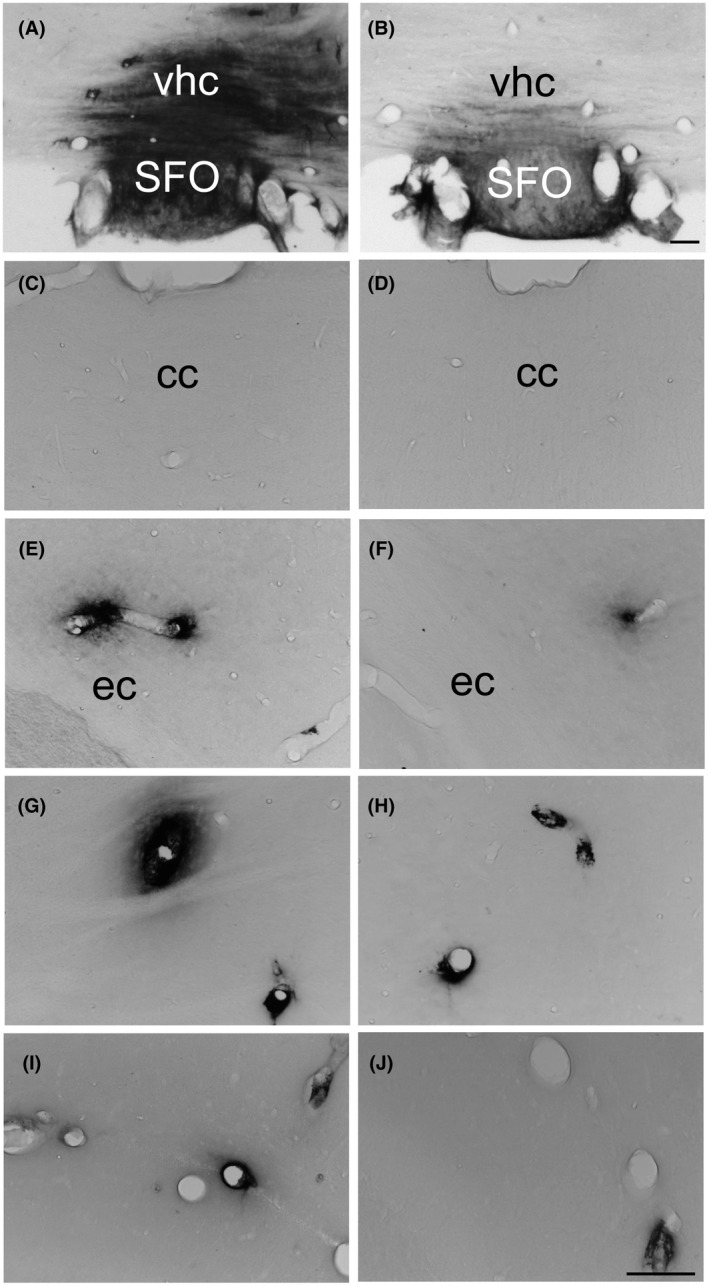
Photomicrographs illustrating the distribution of rat IgG‐immunoreactivity in the subfornical organ and ventral hippocampal commissure (A, B), the corpus callosum (C, D), external capsule (E, F), dorsolateral striatum (G, H) and hippocampus (I, J), 24 h after laparotomy (A, C, E, G, I) or cecal ligature and puncture (B, D, F, H, J) in rats that did not undergo MRI under isoflurane anesthesia. (For illustrations of IgG immunoreactivity in animals that underwent MRI under isoflurane anesthesia, see figure 6.^15^) cc: corpus callosum; ec: external capsule; vhc: ventral hippocampal commissure; SFO: subfornical organ. Arrow heads > and < indicate perivascular diffuse cloud‐like labeling Scale bar = 100 μm

In all animals, IgG was found in brain regions lacking a functional BBB including the choroid plexus, meninges and circumventricular organs from which it spread to surrounding regions (Figure 1A,B). With respect to white matter bundles between bregma +4.85 and −2.85 mm (eg, Figure 1C,D), a two‐way ANOVA did not reveal any significant differences in occurrence and extent of perivascular plume‐like “diffusion clouds” of IgG staining in the corpus callosum and external capsule of animals that underwent imaging under anesthesia as compared to rats that did not undergo MRI (Table 1). A two‐way ANOVA on the occurrence and extent of perivascular plume‐like diffusion clouds of IgG staining in the secondary somatosensory cortex and visceral area of the cortex between bregma +4,85 and −2,85 mm (eg, Figure 1E,F) revealed significant effects of surgery (*F*_1,26_: 11.3, *P* < .01 and *F*_1,26_: 12.1, *P* < .01, respectively; Table 1) and a significant interaction between surgery and imaging under anesthesia (*F*_1,26_: 12.2, *P* < .01 and *F*_1,26_: 5.74, *P* < .05, respectively; Table 1). Post hoc tests showed that perivascular IgG diffusion was less important in the secondary somatosensory cortex and visceral area of the cortex of sham‐operated animals that underwent imaging as compared to sham‐operated rats that were not subject to imaging under anesthesia (*P* < .001 and *P* < .01, respectively; Table 1). Among animals that did not undergo imaging, more IgG diffusion was found in sham‐operated rats as compared to CLP animals in these brain areas (*P* < .001 and *P* < .01, respectively; Table 1). When considering the caudate putamen and the hippocampus (eg, Figure 1G‐J), a two‐way ANOVA on the occurrence and extent of perivascular IgG did not show any significant effects of abdominal surgery or imaging under anesthesia or interactions between these factors (Table 1).

**TABLE 1:** Effects of CLP and MRI under anesthesia on brain perivascular IgG diffusion

Region	Occurrence × extent/section	ANOVA & Post hoc (Benjamini–Hochberg corrected)
SSp	Sh.+: 0.033 ± 0.022; Sh.−: 0.333 ± 0.183 CLP+: 0.061 ± 0.019; CLP−: 0.137 ± 0.066	MRI *P* < .01
SSs	Sh.+: 0.023 ± 0.023; Sh.−: 0.176 ± 0.054 CLP+: 0.027 ± 0.018; CLP−: 0	Surgery *P* < .01; Surgery × MRI *P* < .01 Sh.+ < Sh.− *P* < .001 Sh.− > CLP− *P* < .001
VISC	Sh.+: 0; Sh.−: 0.085 ± 0.043 CLP+: 0.004 ± 0.004; CLP−: 0.019 ± 0.019	MRI *P* <.01; Surgery × MRI *P* < .05 Sh.+ < Sh.− *P* < .01 Sh.− > CLP− *P* < .01
GU	Sh.+: 0.017 ± 0.017; Sh.−: 0.039 ± 0.023 CLP+: 0.003 ± 0.003; CLP−: 0	Surgery *P* < .05
AI	Sh.+: 0; Sh.−: 0.057 ± 0.037 CLP+: 0.011 ± 0.011; CLP−: 0.008 ± 0.008	NS
PIR	Sh.+: 0.026 ± 0.011; Sh.−: 0.106 ± 0.077 CLP+: 0.020 ± 0.009; CLP−: 0.016 ± 0.012	NS
TT	Sh.+: 0.010 ± 0.010; Sh.−: 0.038 ± 0.038 CLP+: 0; CLP−: 0	NS
CP	Sh.+: 0.048 ± 0.017; Sh.−: 0.277 ± 0.157 CLP+: 0.119 ± 0.042; CLP−: 0.028 ± 0.028	NS
GPe	Sh.+: 0; Sh.−: 0.019 ± 0.019 CLP+: 0; CLP−: 0	NS
DG	Sh.+: 0.018 ± 0.018; Sh.−: 0.028 ± 0.028 CLP+: 0; CLP−: 0	NS
cc‐ec	Sh.+: 0.101 ± 0.079; Sh.−: 0.471 ± 0.234 CLP+: 0.142 ± 0.069; CLP−: 0.384 ± 0.135	NS
ec	Sh.+: 0.026 ± 0.015; Sh.−: 0.147 ± 0.086 CLP+: 0.063 ± 0.038; CLP−: 0.141 ± 0.057	NS
fi	Sh.+: 0.116 ± 0.078; Sh.−: 0.059 ± 0.028 CLP+: 0; CLP−: 0.015 ± 0.015	NS

Note

Occurrence multiplied by extension score of perivascular of IgG staining per section for different brain structures. The left column displays brain structures based on Swanson's rat brain atlas,^74^ the middle column shows means ± SEM for the four experimental groups and the right column contains a summary of ANOVA and post hoc tests (when appropriate).

Abbreviations: AI, agranular insular cortex; cc‐ec, corpus callosum‐external capsule transition zone; CLP−, Cecal ligature and puncture‐operated animals that did not undergo MRI under isoflurane anesthesia 24 h later; CLP+, Cecal ligature and puncture‐operated rats that were subject to MRI under isoflurane anesthesia one day later; CP, caudate putamen; DG, dentate gyrus; ec, external capsule; fi, fimbria; GPe, external globus pallidus; GU, gustatory area; NS, nonsignificant; PIR, piriform cortex; Sh−, Sham‐operated animals that did not undergo MRI under isoflurane anesthesia 24 h later; Sh+, Sham‐operated rats that were subject to MRI under isoflurane anesthesia one day later; SSp, primary somatosensory cortex; SSs, secondary somatosensory cortex; TT, tenia tecta; VISC, visceral area.

### 3.3 MRI under anesthesia increases neuronal COX‐2 expression

**FIGURE 2:**
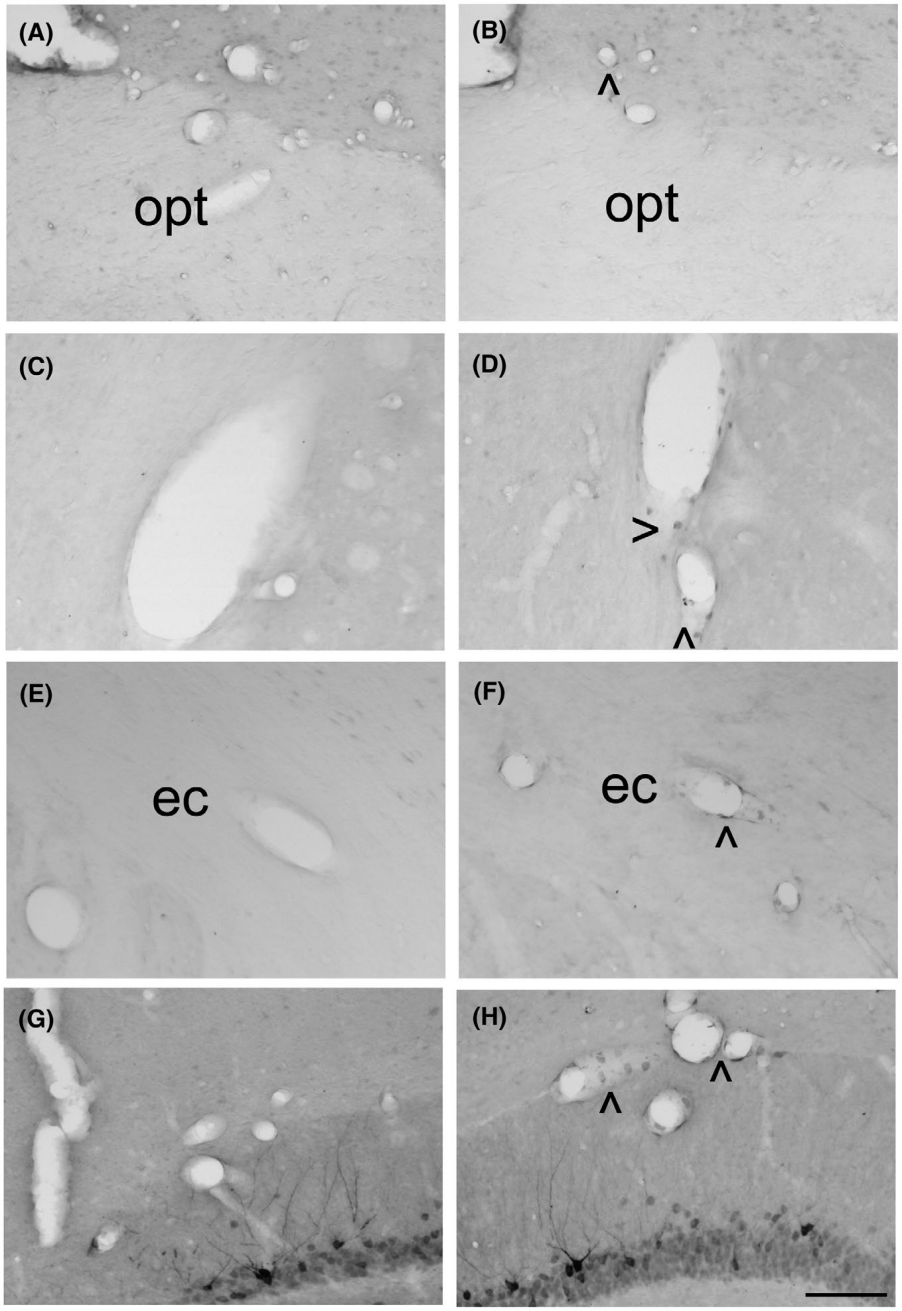
Photomicrographs illustrating the distribution of COX‐2‐immunoreactivity in the ventromedial preoptic area (A, B), caudate putamen (C, D), external capsule (E, F) and hippocampus (G, H) 24 h after laparotomy (A, C, E, G) or cecal ligature and puncture (B, D, F, H) in rats that did not undergo MRI under isoflurane anesthesia. (For illustrations of COX‐2‐immunoreactivity in animals that underwent MRI under isoflurane anesthesia, see figures 3 and 7 of Ref.[15) och: optic chiasm; ec: external capsule. Arrow heads > and < indicate labeling. Scale bar = 100 μm

No or very weak COX‐2 immunoreactivity was found associated with blood vessels in the preoptic area (Figure 2A,B), caudate putamen (Figure 2C,D), external capsule (Figure 2E,F), or hippocampus (Figure 2G,H) of animals subjected to sham surgery. However, distinct perivascular disc‐like COX‐2‐immunoreactive cells were frequently observed in these structures in rats that underwent CLP (Figure 2B,D,F,H).

**FIGURE 3:**
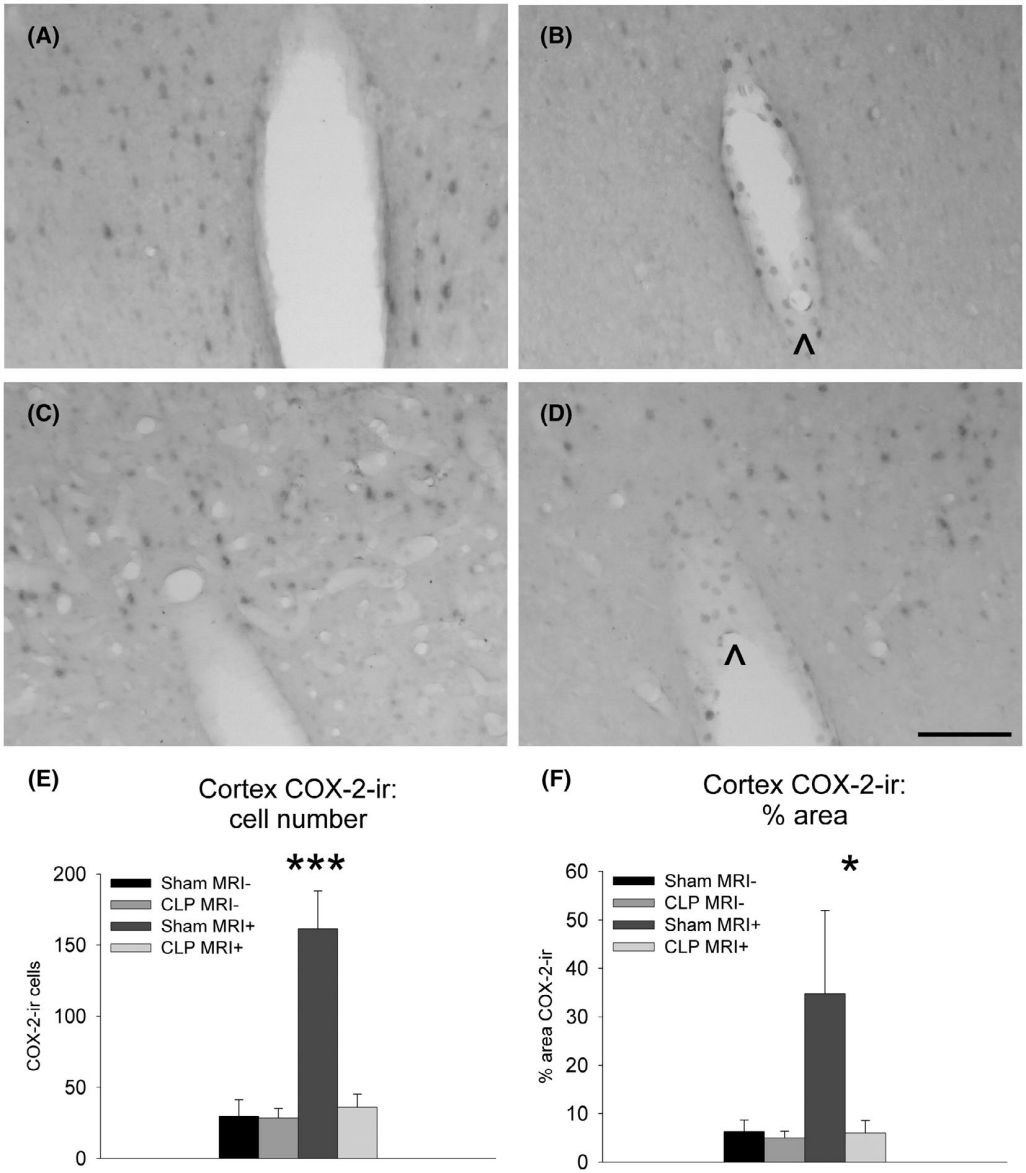
Photomicrographs illustrating the distribution of COX‐2‐immunoreactivity in the cortex of rats that did not undergo MRI under isoflurane anesthesia (A, B) and of animals that were subject to MRI under anesthesia (C, D) 24 h after laparotomy (Sham: A, C) or cecal ligature and puncture (CLP: B, D). Scale bar = 100 μm. Quantification of cortical COX‐2‐immunoreactive cells (E) and surface (F) showing means ± SEM 24 h after cecal ligature (CLP) and puncture or laparotomy (Sham) in animal that underwent or not MRI under anesthesia (MRI±, respectively). ir: immunoreactivity *P < .05. ***P < .001. Group sizes Sham MRI−: n = 4; CLP MRI−: n = 5; Sham MRI+: n = 6; CLP MRI+: n = 7

Constitutive COX‐2 expression was detected in neurons of the hippocampus (Figure 2G,H) and to a lesser extent, in neurons of the cortex (Figure 3A,B). A two‐way ANOVA on the number of COX‐2‐positive elements in the cortex revealed significant effects of abdominal surgery (*F*_1,18_ = 13.7, *P* < .01) and imaging under anesthesia (*F*_1,18_ = 16.6, *P* < .001) as well as a significant interaction between these factors (*F*_1,18_ = 13.2, *P* < .01). Post hoc analysis indicated significant less COX‐2–positive cells in CLP as compared to sham‐surgery in animals that underwent imaging (*P* < .001; Figure 2K) and significantly less COX‐2–expressing cells in sham‐operated rats that did not undergo imaging in comparison to those that did (*P* < .001; Figure 2K). A two‐way ANOVA on the relative surface occupied by COX‐2‐immunoreactivity in the cortex indicated a significant effect of abdominal surgery (*F*_1,18_ = 4.86, *P* < .05) and trends for an effect of imaging under anesthesia (*F*_1,18_ = 3.69, *P* = .071) as well as for an interaction between factors (*F*_1,18_ = 3.85, *P* = .066). Post hoc analysis showed a significantly lower relative surface of COX‐2‐immunoreactivity in the cortex of animals that underwent CLP as compared to sham surgery (*P* < .05; Figure 3E) as well as a significantly lower relative surface of COX‐2‐immunoreactivity in sham‐operated rats that did not undergo imaging in comparison to those that did (*P* < .05; Figure 3F).

### 3.4 MRI under anesthesia and sepsis alter corpus callosum microglia

**FIGURE 4:**
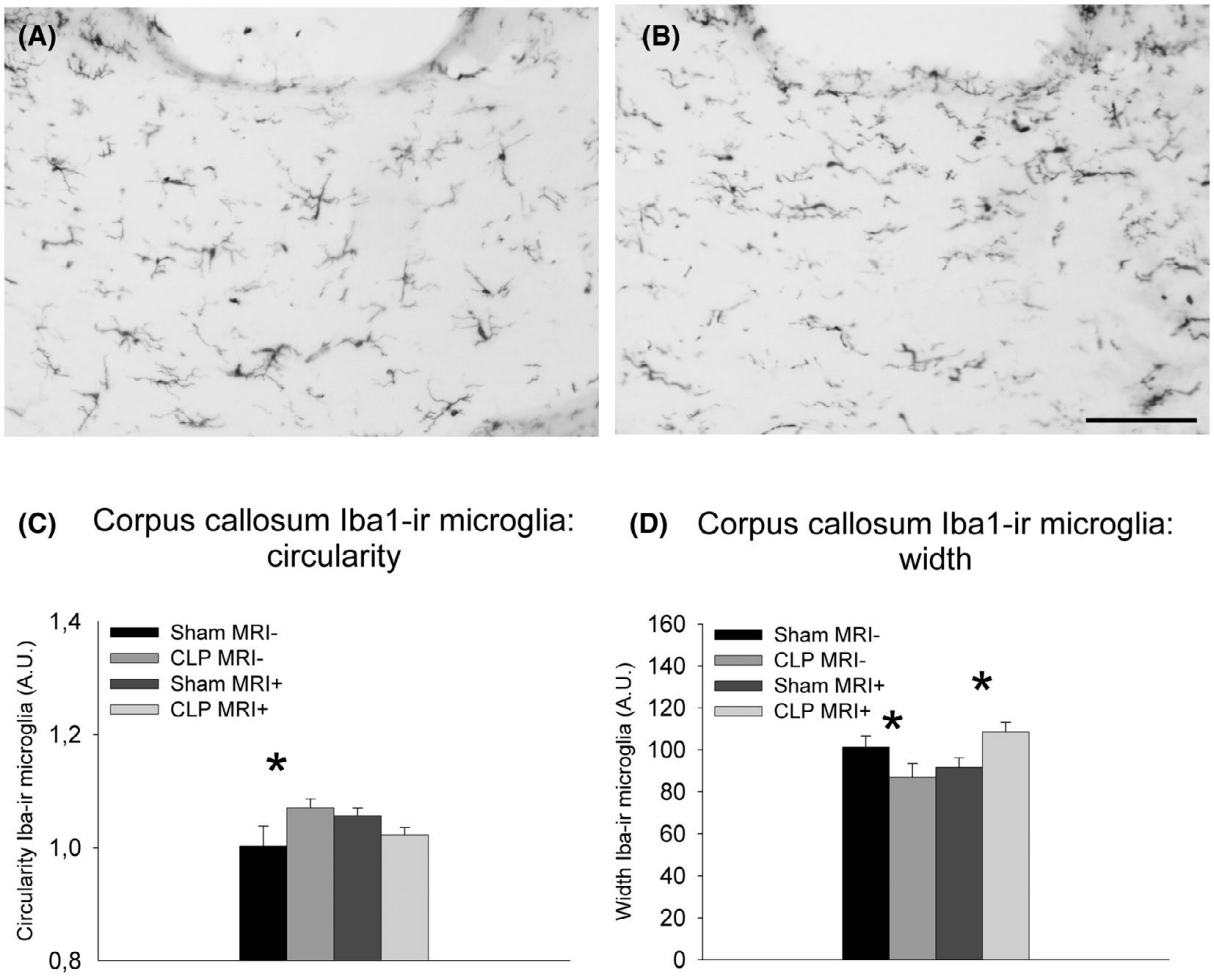
Photomicrographs illustrating Iba1‐ir microglia in the corpus callosum after laparotomy (A) or cecal ligature and puncture (B) in rats that did not undergo MRI under isoflurane anesthesia. (For illustrations of Iba‐1‐immunoreactivity in animals that underwent MRI under isoflurane anesthesia, see figure 8.^15^) Quantitative Fraclac‐based analysis of microglial circularity (C) and width (D) in the corpus callosum showing means ± SEM for the four experimental groups. ir: immunoreactivity *P < .05. Group sizes Sham MRI−: n = 4; CLP MRI−: n = 4; Sham MRI+: n = 9; CLP MRI+: n = 11. Scale bar = 100 μm

Two‐way ANOVAs on the number of and relative surface occupied by Iba‐1–positive elements (Figure 4A,B) in the corpus callosum between bregma −0.11 and −1.33 mm with surgery and imaging under anesthesia as between factors did not indicate any significant differences. Similar analyses on cell and cell body sizes and activation state of Iba‐1–stained microglia according to Hovens et al^31^ revealed a trend for an effect of imaging under anesthesia on cell size (*F*_1,23_ = 3.00, *P* = .096) and a trend for an interaction between surgery and imaging under anesthesia for cell size (*F*_1,23_ = 3.34, *P* = .081) and activation state (*F*_1,23_ = 3.18, *P* = .09).

Two‐way ANOVAs on fractal parameters of Iba‐1–positive cells in the corpus callosum between bregma −0.11 and −1.33 mm revealed a significant interaction between abdominal surgery and imaging under anesthesia for circularity (*F*_1,24_ = 7.00, *P* < .05) and cell width (*F*_1,24_ = 7.32, *P* < .05) and a trend for an interaction between treatments for cell height (*F*_1,24_ = 3.08, *P* = .091). Post hoc analyses indicated a significantly increased circularity in CLP compared to sham surgery in animals that did not undergo imaging under anesthesia (*P* < .05; Figure 4C) and trends for increased circularity in sham‐operated animals that underwent imaging under anesthesia as compared to sham‐operated rats that did not (*P* = .062) as well as for decreased circularity in CLP rats that were imaged as compared to CLP animals that were not (*P* = .088). In addition, post hoc analyses showed a significantly lower width in CLP rats that did not undergo subsequent imaging in comparison to CLP animals that were next imaged under anesthesia (*P* < .05; Figure 4D) as well as significantly higher width after CLP than after sham surgery in those animals that underwent imaging (*P* < .05; Figure 4D).

### 3.5 MRI under anesthesia alters corpus callosum astrocytes

**FIGURE 5:**
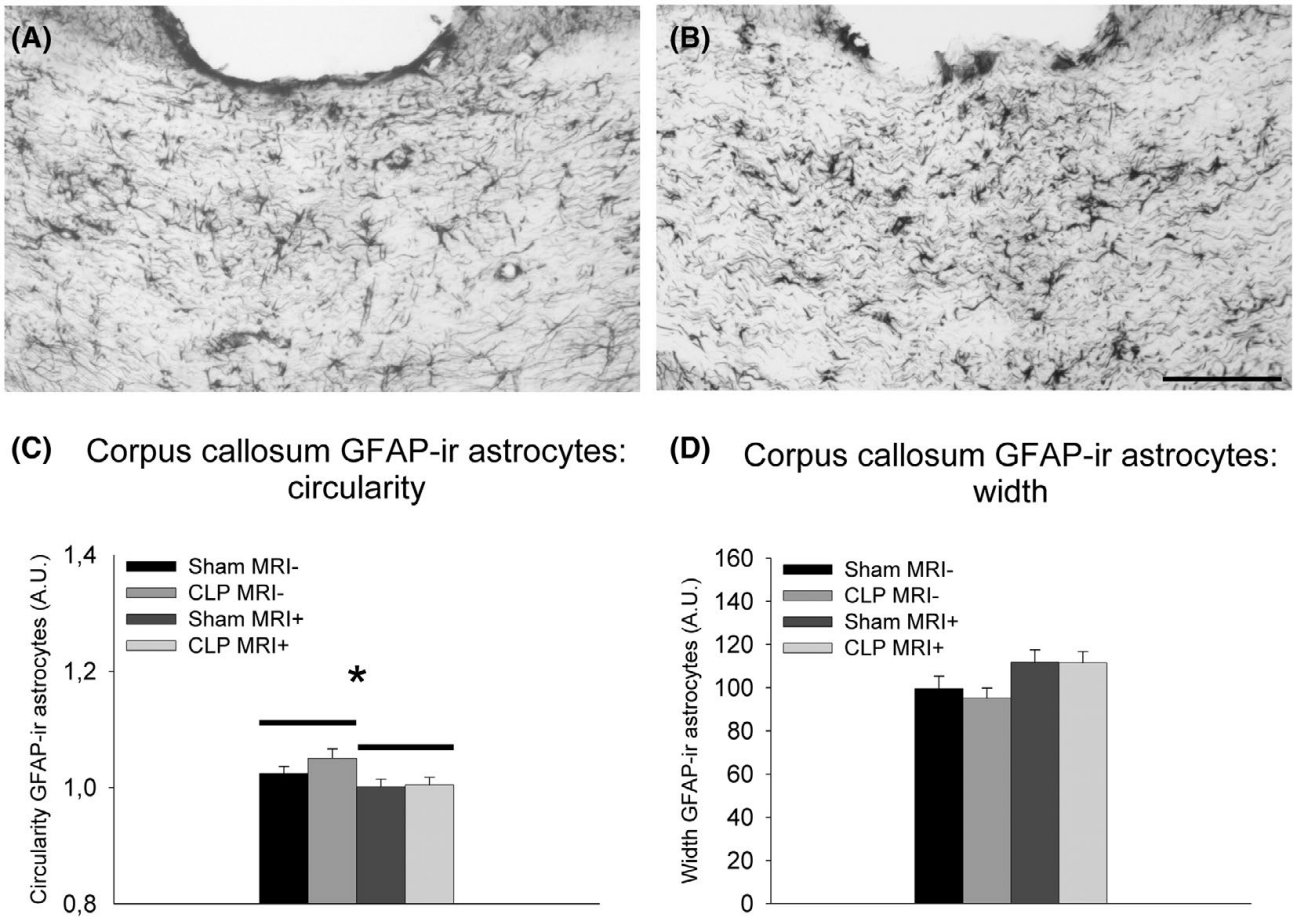
Photomicrographs illustrating GFAP‐ir astrocytes in the corpus callosum after laparotomy (A) or cecal ligature and puncture (B) in rats that did not undergo MRI under isoflurane anesthesia. (For illustrations of GFAP‐immunoreactivity in animals that underwent MRI under isoflurane anesthesia, see figure 9.^15^) Quantitative analysis of Fraclac‐based astrocyte circularity (C) and width (D) in the corpus callosum showing means ± SEM for the four experimental groups. ir: immunoreactivity *P < .05. Group sizes Sham MRI−: n = 4; CLP MRI−: n = 4; Sham MRI+: n = 9; CLP MRI+: n = 11. Scale bar = 100 μm

Two‐way ANOVAs on the number of and relative surface occupied by GFAP‐positive elements (Figure 5A,B) in the corpus callosum between bregma −0.11 and −1.33 mm with surgery and imaging under anesthesia as between factors revealed no significant differences. Similar analyses on fractal parameters of GFAP‐positive cells in the corpus callosum between bregma −0.11 and −1.33 mm indicated that imaging under anesthesia significantly decreased circularity (*F*_1,24_ = 6.04, *P* < .05; Figure 5C) as well as trends for imaging under anesthesia to increase cell perimeter (*F*_1,24_ = 3.75, *P* = .065) and cell width (*F*_1,24_ = 3.82, *P* = .062; Figure 5D).

## 4 DISCUSSION

The main findings of this study were that MRI under isoflurane anesthesia one day after abdominal surgery reduced brain perivascular diffusion of IgG, altered white matter astrocyte morphology, and increased cortical neuronal COX‐2 immunoreactivity. In addition, it annihilated CLP‐induced increased circularity of white matter microglial cells, but did not affect CLP‐induced perivascular COX‐2 expression. Thus, these findings indicate that MRI performed under anesthesia in rodents alters results obtained with histological approaches during neuroinflammation secondary to sepsis.

The loss of the righting reflex in animals that underwent CLP indicated brain dysfunction, as previously shown.^28^, ^32^, ^33^ Even though sham‐operated animals did show a slight transient decrease in the reflexes studied during the first hours after anesthesia, the effects of CLP were much more pronounced and lasted at least up to a day for the righting reflex.^15^ Interestingly, repeated exposure to isoflurane anesthesia at a 3‐day interval has been shown to alter rodent behavior,^34^ while several episodes of isoflurane anesthesia at 10 day intervals have been reported to affect behavior much less.^35^ Thus, for behavioral assessments it seems important to either perform them after a sufficiently longer recovery period after either one or several episodes of isoflurane anesthesia.

Somewhat surprisingly, perivascular diffusion of IgG was less important in the cortex of rats that underwent 3 hours of MRI under isoflurane anesthesia 1 day after sham surgery as compared to those that underwent abdominal surgery without subsequent imaging under anesthesia. As brain MRI in animals typically involves both exposure to magnetic fields and radiofrequencies as well as anesthesia, it is important to consider both factors when trying to make sense of the findings obtained in the present work. Studies have shown effects of exposure to 1.5‐3.0 T magnetic fields on human lymphocytes.^36^, ^37^ The evidence regarding the effects of MRI‐related electromagnetic waves on the BBB is conflicting and conclusions are hard to drawn because of the many different frequencies used and potential confounders.^38^ Interestingly, it has been reported that important changes in magnetic field can actually entail a decrease in BBB permeability of pentobarbital‐anesthetized rats to a 1.5 T magnetic field.^39^ As animals that underwent MRI under anesthesia in the present work were subject to echo planar imaging pulse sequences that involve rapidly changing magnetic fields,^15^ this may explain why indications of BBB breakdown were less frequently observed in the cortex of animals on which abdominal surgery was performed before being imaged than in those that just underwent laparotomy without subsequent MRI.

Compared to the effects of MRI on brain structure and function, those of isoflurane anesthesia have been studied much more extensively. In the present study, perivascular diffusion of IgG was occasionally found in the cerebral cortex as well as in the hippocampus, striatum, and white matter of rats that underwent sham surgery under 1 hour of isoflurane anesthesia the day before. This observation may be explained by the finding that intravascularly injected fluorescent dextran can still be detected in the brain, thus indicating BBB breakdown, 17 hours after laparotomy under isoflurane anesthesia.^40^ Although a previous study has shown that 3 hours of isoflurane anesthesia alone is not sufficient to alter the frequency or extent of perivascular diffusion of IgG in the cortex of rats 24 hours later,^12^ brain sections in that study were not stained with Ni‐enhanced diaminobenzidine revelation of antibody‐linked peroxidases and may therefore have resulted in less contrast as compared to our staining protocol. In addition, in the present work, perivascular diffusion of IgG was found to be less important in the cortex of animals that underwent 3 hours of MRI under isoflurane anesthesia 1 day after sham surgery as compared to those that underwent abdominal surgery without subsequent imaging under anesthesia. Since re‐exposure to isoflurane or sevoflurance anesthesia at a 24‐hour interval has been shown to have neuroprotective effects and to preserve BBB integrity,^41^, ^42^, ^43^ it is hypothesized that the two episodes of isoflurane anesthesia, first for abdominal surgery and then for MRI, resulted in protection against the effects of a single exposure to anesthesia on the BBB.

Although BBB breakdown has been proposed to play a role in brain dysfunction during sepsis,^44^ our previous work using CLP followed by brain imaging and histology, did not show increased perivascular diffusion of IgG, except in the fimbria.^15^ As this was at variance with numerous previous studies indicating BBB breakdown after CLP, but that did not use imaging,^45^, ^46^, ^47^, ^48^, ^49^, ^50^ we wondered if MRI under isoflurane anesthesia played a role in our previous finding. However, assessment of BBB integrity with IgG detection on brain sections in animals that had not undergone MRI confirmed that CLP, as compared to sham surgery, did not increase the frequency and extent of perivascular IgG diffusion in the different forebrain regions examined. It is important to keep in mind here that the severity of CLP‐induced sepsis depends on needle size and on the number of punctures^51^ and that the needle size used here was smaller than those employed in previous work reporting BBB breakdown after CLP in rodents.^45^, ^46^, ^47^, ^48^, ^49^, ^50^ Hence, our experimental model induces signs of neurological impairment in the absence of widespread BBB breakdown for molecules of high molecular weight.

Interestingly, it was even observed in some cortical structures that the extent of perivascular IgG diffusion, even though overall low, was even lower 24 hours after CLP as compared to sham surgery in animals that did not undergo MRI under isoflurane anesthesia. As astrocytic endfeet rapidly swell along with perivascular microedema after fecal peritonitis or CLP,^52^, ^53^, ^54^, ^55^, ^56^ this may have limited the extent of perivascular IgG diffusion. Since the increased T2‐weigted intensities in the cortex and striatum after CLP, as found in our previous work, can be interpreted to suggest the presence of more water,^15^ this may explain the lesser extent of perivascular IgG diffusion 24 hours after CLP as compared to sham surgery.

Glia cell dysfunction has also been proposed to play a role in sepsis‐associated brain dysfunction.^44^ As isoflurane affects the cytoskeleton of rat astrocytes in vitro,^57^ the effects of MRI under isoflurane anesthesia on the astrocytic cytoskeleton intermediate filament marker GFAP were studied in rats that had undergone laparotomy or CLP 24 hours earlier. MRI under anesthesia 24 hours after laparotomy decreased circularity of GFAP‐stained astrocytes in the corpus callosum. Interestingly, reactive astrocytes in white matter show hypertrophy of cell bodies, retraction of processes and a reduction of branching, and loss of their orientation,^58^ which can be expected to result in increased circularity. Our findings can thus be interpreted to suggest that MRI under anesthesia could prevent activation of white matter astrocytes.

As isoflurane attenuates the increased expression of the microglial marker Iba‐1 after subarachnoid hemorrhage,^59^ it was of interest to study if MRI under isoflurane anesthesia influences brain Iba‐1 expression after CLP. Interestingly, these analyses revealed increased circularity of Iba‐1–positive cells in the corpus callosum after CLP compared to sham surgery in animals that did not undergo MRI under anesthesia and decreased circularity of microglia in CLP rats that were imaged as compared to CLP animals that were not. Since increased circularity is a hallmark of activation of microglia,^60^, ^61^, ^62^ our findings indicate that MRI under anesthesia masked CLP‐associated microglial activation.

The prostaglandin‐synthesizing enzyme COX‐2 mediates neurovascular coupling^29^, ^30^ and prostaglandins have been proposed to play a role in isoflurane‐induced hyperemia.^63^ Since our previous work indicated both lower blood distribution and decreased neuronal COX‐2 in the cortex of CLP rats that underwent MRI under anesthesia,^15^ it was important to study if the latter effect was also observed without imaging under isoflurane anesthesia. MRI under anesthesia increased the number of COX‐2–positive cells in the cortex in particular in animals that underwent sham surgery. Interestingly, the decrease in the number of COX‐2–positive elements in the cortex of CLP rats as compared to sham‐operated animals that underwent MRI imaging under isoflurane anesthesia was not observed in rodents that did not undergo imaging. Regarding this finding, it is important to note that surgery on other body parts than the abdominal cavity under isoflurane anesthesia also increases COX‐2‐immunoreactivity in neurons of the spinal cord.^64^ Together, these findings suggest that isoflurane increases neuronal COX‐2 throughout the CNS. Of note, the perivascular induction of COX‐2 after CLP was not altered by MRI under anesthesia.

There are potentially important implications of the present findings. One may wonder if the reduced glial activation in animals that were subject to MRI under isoflurane anesthesia may have influenced the increased water diffusion parallel to white matter fibers after CLP was found with diffusion‐weighted imaging.^15^ Nevertheless, it has been shown that peritonitis disrupts the tubulin network inside white matter axons in rodents.^65^ It is therefore likely that this would still result in changes in diffusion imaging. Interestingly, the detection of neurofilaments in blood is used both in humans and animals as a way to monitor neuronal damage.^65^, ^66^, ^67^, ^68^, ^69^ In this context, it is important that general anesthesia alters blood neurofilament concentrations already in neurologically healthy individuals.^70^, ^71^ Moreover, given that isoflurane anesthesia in rodents slows down brain drainage via the cerebrospinal fluid,^72^, ^73^ this may result in an underestimation of neurofilament disruption occurring in the brain parenchyma.

In conclusion, this study showed that MRI under isoflurane anesthesia reduced BBB breakdown, decreased circularity of white matter astrocytes, and increased neuronal COX‐2 immunoreactivity in the cortex of rodents 24 hours after laparotomy. In addition, it annihilated CLP‐induced increased circularity of white matter microglia and astrocytes. MRI under isoflurane anesthesia did, however, not alter sepsis‐associated perivascular COX‐2 induction. These effects may be related to re‐exposure of the animals to isoflurane anesthesia between abdominal surgery and MRI. As there seem to be no real alternatives, the best the scientific community can do is to be aware of these effects and to not suppose that because a procedure is done under isoflurane anesthesia or is noninvasive it would not affect the subject of interest. The present findings indicate that MRI under isoflurane anesthesia of rodents can modify neurovascular coupling and glial activation and should, therefore, be interpreted with caution, especially in the context of translational research.

## AUTHOR CONTRIBUTIONS

ID performed experiments and data analysis, wrote the first version of the manuscript as well as edited and approved of the manuscript; MG performed experiments and data analysis, and edited and approved of the manuscript; JPK designed and performed experiments as well as data analysis and wrote the final manuscript.
